# Mammographic Assessment of a Geographically Defined Population at a Mastology Referral Hospital in São Paulo Brazil

**DOI:** 10.1371/journal.pone.0074270

**Published:** 2013-09-16

**Authors:** Simone Caetano, Juvenal Mottola Junior, Flora Finguerman, Suzan M. Goldman, Jacob Szejnfeld

**Affiliations:** 1 Fundação de Pesquisa e Estudo de Diagnóstico por Imagem (FIDI), São Paulo, Brazil; 2 Secretaria de Saúde do Estado de São Paulo, São Paulo, Brazil; 3 Departamento de Diagnóstico por Imagem, Universidade Federal de São Paulo-Escola Paulista de Medicina, São Paulo, Brazil; Faculty of Medicine, University of Porto, Portugal

## Abstract

**Objective:**

To evaluate the results of screening and diagnostic mammography in a geographically defined population attending a regional mastology referral hospital of the State Public Service of São Paulo.

**Methods:**

A total of 7508 women, who received screening or diagnostic mammography examinations from 06/2004 to 06/2005, with follow-up until 06/2006, were included in this study. Data corresponding to age, the Breast Imaging-Reporting and Data System (BI-RADS), biopsy, surgery and the stage of breast cancer were collected. Five-year survival of patients with breast cancer was posteriorly calculated during this period.

**Results:**

This study included a total of 713 diagnostic and 6795 screening mammograms. The average age of the population was 51.2 years, with a BI-RADS end result of 4 and 5 (abnormal) in 1.9% of the screening and 11.4% of the diagnostic mammograms, respectively. All BI-RADS category zero was complemented. Of the 228 nonsurgical biopsies performed (71 CNB, 94 mammotomy and 63 FNAB), 63 (27.6%) biopsies were malignant findings. Among the 33 surgical biopsies, 10 (30.3%) biopsies were malignant findings, and of the 82 surgeries, 55 (67, 1%) procedures showed malignant findings. Seventy-one (0.9%) breast cancers (25/6795 on screening exams and 46/713 on diagnostics) were diagnosed. A total of 28.6% small cancers (≤10 mm) were observed, with 27% of the cancers in stages zero and I. Approximately 47.6% of the cases showed nodal invasion, and 4.5% of cases were not staged. Overall detection rate of breast cancer was 8.8/1000 (3.2/1000 screening and 61.7/1000 diagnostic). The overall 5-year survival rate of patients with breast cancer in this population was 79.1%.

**Conclusion:**

Survival is a key index of the overall effectiveness of health services in the management of patients with cancer. Our results suggest that this approach is feasible and can potentially improve breast cancer outcomes for many women in São Paulo.

## Introduction

Breast cancer is one of the leading causes of death worldwide, remaining the second most common type of cancer among women, with the highest mortality rate among all cancers in America and western North America. In Canada and the United States, the statistics show that one in ten women will develop cancer during her lifetime [1], [2]. As the second most prevalent cancer worldwide and the most common cancer among women, breast cancer accounts for 22% of new cases each year. If diagnosed and treated early, the prognosis is more favorable [1], [2].

In general, incidence rates are high in Western and Northern Europe, Australia/New Zealand, and North America, while intermediate rates have been observed in South America, the Caribbean, and Northern Africa, and low rates occur in sub-Saharan Africa and Asia. The factors that contribute to the international variation in incidence rates reflect differences in reproductive and hormonal factors and the availability of early detection services. Reproductive factors that increase the risk of breast cancer include a long menstrual history, nulliparity, recent use of postmenopausal hormone therapy or oral contraceptives, and late age at first birth. Alcohol consumption also increases the risk of breast cancer [2].

Even if relatively rare before age 35, the incidence of breast cancer increases rapidly and progressively above this age group. Statistics show an increased frequency in both developed and developing countries. According to the World Health Organization (WHO), in the 60's and 70’s a ten-fold increase in age-adjusted incidence rates was recorded in population*-*based cancer registries from different continents [1], [2].

In Brazil, the mortality rates for breast cancer remain high, potentially reflecting disease diagnosis at advanced stages. In the world population, the average 5-year survival rate is 61% [1].

In São Paulo, the presumed prevalence of suspected and highly suspected breast cancer lesions from mammograms was 0.615% in 2002 [3].

World screening projects were established approximately 40 years ago, with variations in the age of patients and the screening approach used. The results of these programs reflect a significant reduction of mortality from breast cancer [4]–[6].

Tabar et al. confirmed a reduction of 31% in mortality from breast cancer and a 25% reduction in stage II or more advanced cancer diagnoses, reflecting the introduction of a mass screening program in Sweden in 1977 [7]. Currently, the screening program of the American College of Radiology (ACR) recommends annual mammography in patients older than 40 years [8]. In patients less than 40 years of age, mammography is recommended either when the patients are considered high risk for developing the disease or to investigate a palpable breast mass [6], [9].

After the introduction of mammography screening programs, a reduction of 25–35% in mortality from breast cancer in women from 50 to 74 years of age and a 10–18% reduction in women from 40 to 49 years of age were observed [10], [11]. In April 2004, screening mammography was established in Brazil for women between 50 and 69 years of age, with a maximum two-year interval between examinations [1].

The BI-RADS system was applied to classify breast lesions. In 1992, the American College of Radiology (ACR) and the Brazilian Society of Mastology (SBM) developed this system to standardize mammographic reports. Since 1998, this system has been used in Brazil, according to the guidelines of the Brazilian College of Radiology (CBR) and the Brazilian Federation of Gynecology and Obstetrics (FEBRASGO). Thus, assessing the effectiveness of the use of the BI-RADS system is important for the early detection of breast cancer.

In April 2004, the Breast Health Program of the Northern Region in the General Hospital in Vila Nova Cachoeirinha (HGVNC) "Dr Álvaro de Souza Simões" was initiated through the State Health Department of São Paulo to provide a specialized reference for breast diseases, regarding the diagnosis, treatment, rehabilitation and follow-up of a target population. This population, including Basic Health Units in São Paulo districts of Vila Nova Cachoeirinha, Brasilândia, Freguesia de Ó, Casa Verde and Limão, comprised 29 units referred to the program based on the condition that each unit followed the established protocols between HGVNC (Referral Hospital) and the referred basic health units. A protocol was created for the participating patients with and without symptoms; specific lessons were administered for the orientation of health professionals of the region and talks were held to raise the awareness of the local population.

In the HGNVC diagnostic mammography service, only the districts of Freguesia do Ó and Brasilândia were assisted, resulting in a total target population of approximately 58,467 women over 40 years (census from 2000).

Considering these aspects, it can be inferred that the HGVNC presents a regionalized and specialized service for breast health, intended for a geographically defined population of the city of São Paulo. Thus, due to the lack of epidemiological data concerning this disease in Brazil, the aim of this study was evaluate this service.

### Objectives

The objective of this study was to evaluate the results of screening and diagnostic mammography in a geographically defined population of patients from a regionalized mastology referral hospital of the Public Service of São Paulo, using measures of association, including sensitivity and specificity, positive predictive values, mammography recall rates, breast cancer detection rates and the survival study of breast cancer patients diagnosed during this period.

## Methods

### Ethics Statement

This study was reviewed and fully approved by the Research Ethics Committee of the Universidade Federal de São Paulo-Escola Paulista de Medicina (UNIFESP-EPM) (0919/07). The IRB granted a waiver of written consent due to the retrospective nature of this study

### The Mastology/Breast Imaging Diagnostic service in the Woman Health Sector of the General Hospital of Vila Nova Cachoeirinha

In April 2004, the Breast Health Program of the Northern Region in the General Hospital of Vila Nova Cachoeirinha (HGVNC) "Dr Álvaro de Souza Simões" was initiated through the Department of Health of the State of São Paulo to provide a specialized reference for breast diseases, regarding the diagnosis, treatment, rehabilitation and follow-up of a target population of Basic Health Units in São Paulo, from the districts of Vila Nova Cachoeirinha, Brasilândia, Freguesia de Ó, Casa Verde and Limão, comprising a total 29 units referred to the program, meeting the established protocols of the HGVNC (Referral Hospital) and the referred basic health units. A protocol was created for treating symptomatic and asymptomatic patients; specific talks were held to increase the awareness of the local population and lessons were administered for the orientation of health professionals of the region.

In the case of HGNVC diagnostic mammography service, only the districts of Freguesia do Ó and Brasilândia were assisted, totaling a target population of approximately 58,467 women over 40 years (2000-IBGE census).

The patients attended the referenced Basic Health Units (BHU) (Freguesia do Ó and Brasilandia) and were referred to the Department of Diagnostic Imaging Breast of the HGVNC to perform mammography. The symptomatic patients were referred directly to the Department of Mastology at the hospital before performing the mammography.

A multidisciplinary team (breast oncologists and radiologists) determined the protocol for obtaining a positive diagnostic mammography (routine follow-up or surgery).

In tests with benign lesions (BI-RADS categories 1 and 2), routine examinations were suggested, and the patients were referred for care at the initial Basic Health Unit. In patients with benign findings (BI-RADS category 3), follow-up was suggested at 6, 12 and 24 months; the patients were monitored in the Basic Health Unit. In cases where the patients had a family history (first degree relatives), a biopsy was recommended. The suspected cases (BI-RADS categories 4 and 5) were referred to the Ambulatory Center of Radiology-Mastology Resolution and Integration to undergo procedures for definitive diagnosis. When the outcome of these exams was benign, the patients were discharged with new references to the Basic Health Unit. When malignancies were detected, the patients were referred to the Mastology service to continue the protocols. Suspicious or inconclusive cases after biopsies continued follow-up in the Mastology service at the hospital. In inconclusive cases (BI-RADS category zero), complementary diagnostic exams were encouraged (additional mammography or ultrasound), returning the patient to the Basic Health Unit and rescheduling complementary exams at the department of Diagnostic Breast Imaging. In these cases, the attending physician at the Basic Health Unit performed the complementary exams. In most of inconclusive cases (BI-RADS category zero), the radiologist performed exams using bright light radiology, with additional views, or even ultrasound.

### Evaluation of the exams

A team of seven radiologists, with at least 4 years of experience in breast imaging, analyzed the mammograms. Each exam was standardized according to the BI-RADS method. [9] These radiologists also supervised the quality standard of the examination. In addition, through a weekly program, a specialized team of technicians developed and fixed the films. Qualified physicists performed the periodic control of the image quality.

A conventional mammography screening technique, comprising four mediolateral oblique and craniocaudal views bilaterally, using a Siemens ® Mammomat 3000 equipped with an analog system, was employed. In some cases, additional images were captured according to the indication of the present radiologist.

The mammograms were classified as diagnostic (women with clinical breast complaints) and screening (asymptomatic), according to the technical questionnaire collected during the exam.

The positive mammograms were classified as BI-RADS categories 0 (zero), 4 and 5. All mammograms BI-RADS category 0 (zero) were reclassified after adequate complementation and the final BI-RADS obtained (additional mammographic or ultrasound device on Toshiba ® Nemio).

In cases requiring investigations through anatomopathological analysis, the material was obtained using fine-needle aspiration (FNAB), core-needle biopsy (CNB) or thick-needle vacuum-assisted biopsy (Mammotome Endo-Surgery Inc. ® Johnson and Johnson company) and submitted to the pathology laboratory, which specializes in breast cancer. In cases where the material was inadequate, a second collection was performed. All biopsies were catalogued for later reference.

### Sample population

#### Inclusion criteria

- Women with positive mammogram (BI-RADS categories 4 and 5) and cytopathological data were followed in the service; patients without cytopathological data were subjected to a mammographic follow-up for a minimum of 24 months;

- Women with a negative test (BI-RADS categories 1, 2 and 3) were subjected to a mammogram follow-up for 12 consecutive months.

#### Exclusion criteria

- Women with no follow-up during the 12 consecutive months after a negative mammogram;

- Women without cytopathological data after positive mammography or without a minimum mammography follow-up for 24 months;

- Mammogram BI-RADS category 6 was excluded (three exams).

A total of 7508 women who underwent mammography in the diagnostic imaging service at General Hospital of Vila Nova Cachoeirinha from June 2004 to June 2005 met the inclusion criteria for this study. The subjects were obtained from two health centers (Freguesia de Ó and Brasilândia) of the North Zone of São Paulo (18 UBS), and 1% of the subjects were obtained elsewhere.

These regions are characterized by low socioeconomic status with high infant mortality (13.65/1,000 live births). A significant percentage of the inhabitants lived in slums (13.23% versus 11.12% in the city of Sao Paulo), with an annual growth of 3.68%. The average household income of this population was approximately $ 390 USD, while in the city of Sao Paulo the average income is $ 663 USD, with a high prevalence of people under 40 years of age (insert reference here).

### Study variables

The variables were obtained through a database service, stored in "CDI examinations" software, which uses Microsoft Office Access ®, the medical records from patients who underwent procedures and surgeries and the results of the biopsies performed.

The cancer staging and diagnosis was determined based on the classification of malignant tumors (TNM), proposed by the International Union Against Cancer (UICC), according to the dimensions of the primary tumor, involvement of the lymph nodes in the lymphatic drainage chain of the breast and the presence or absence of distant metastases.

The numbers of diagnostic and screening mammography and their classifications were collected using BI-RADS; true positives (TP), true negatives (TN), false negatives (FN), false positives (FP), the total number of cancers in the sample, and the cancer staging were determined; the biopsies, surgeries and surgical outcomes were also documented.

### Statistical Analysis

The outcome variable of the study was positive for cancer in the cytological or histopathological samples, fragment biopsies and surgical specimens.

The statistical analysis was performed using a database in Microsoft Office Excel®, and the analyses were performed using the public domain software Epi Info 7.

The measured parameters and methods to estimate the performance of the service are described in the auditing section of the 4th edition of the ACR-BI-RADS (9).

The true-positive exams were defined as exams with a positive interpretation, followed by the diagnosis of invasive breast carcinoma or ductal carcinoma *in situ* within 12 months. The rate of cancer detection was defined as the number of cancers (positive exam) divided by the total number of exams. One false positive was defined as a positive interpretation with no cancer diagnosis after follow-up.

We calculated the positive predictive value (PPV) by dividing the number of true positive tests by the total number of tests (true positive and false positive tests). Three separate PPV were calculated using the BI-RADS method: PPV1 (probability of cancer after an examination with initial positive BI-RADS categories zero, 4 and 5), PPV2 (probability of cancer, followed by final BI-RADS categories 4 and 5), and PPV3 (probability of cancer in biopsied patients after final BI-RADS categories 4 and 5, also known as a positive biopsy rate). For mammograms with an initial BI-RADS category 0 (zero), the final BI-RADS category was determined after performing additional images or ultrasound. The final classification was used to calculate VPP2 and VPP3. The tests with final BI-RADS categories 4 and 5 were recommended for biopsies. All biopsy types were included in VPP3 (FNAG, CNB, mammotomy or surgical biopsies).

False negatives were defined as exams with an initial negative result (BI-RADS category 1, 2 and 3) and a diagnosis of invasive breast carcinoma or ductal carcinoma *in situ* within 12 months. True negatives were considered as exams with negative initial interpretations and subsequent negative tests in 12 months.

Simple descriptive statistics were determined (frequency counts, percentage, mean and median).

During this period, the survival time (in months) of patients diagnosed with breast cancer was defined as the period between the date of diagnosis and the last medical consultation or the occurrence of death attributed to breast cancer, according to the death certificate. At the end of the study, all patients with no record of death, because they were still alive or died of different causes, contributed to the follow-up time for the group risk composition.

The TNM system was used for tumor staging. The tumor size was grouped into three ranges: less than or equal to 2 cm (T1), 2–5 cm (T2) and larger than 5 cm (T3). The involvement of lymph nodes was ranked N0 (no lymph node metastasis), N1 (metastasis to mobile ipsilateral axillary lymph nodes) and N2 (metastasis to axillary lymph nodes fixed to one another or structures). Metastasis was classified as M0 (no distant metastases) or M1 (presence of distant metastases).

The Kaplan-Meier method was used to estimate the probabilities of survival.

## Results

### Age groups and BI-RADS

Among the mammographies performed, 713 (9.5%) exams were diagnostic and 6795 (90.5%) exams were screening mammographies.

The mean age of the study population was 51.2 years (SD 10.5) with a median age of 50.5 years, ranging between 15 and 99 years.

In the screening mammography (6795), the average age of the women was 52.2 years (SD 9.8), with a median age of 50.5 years, ranging between 36 and 99 years. In the diagnostic mammography (713), the average age was 42.2 years (SD 11.8), with a median age of 38 years, ranging between 15 and 87 years.

Of the 7508 women subjected to mammography, 71 (0.9%) women had breast cancer, 0.4% (25 of 6795) of these women underwent screening mammographies and 6.5% (46 of 713) of these women were subjected to diagnostic mammographies.

**Figure 1:**
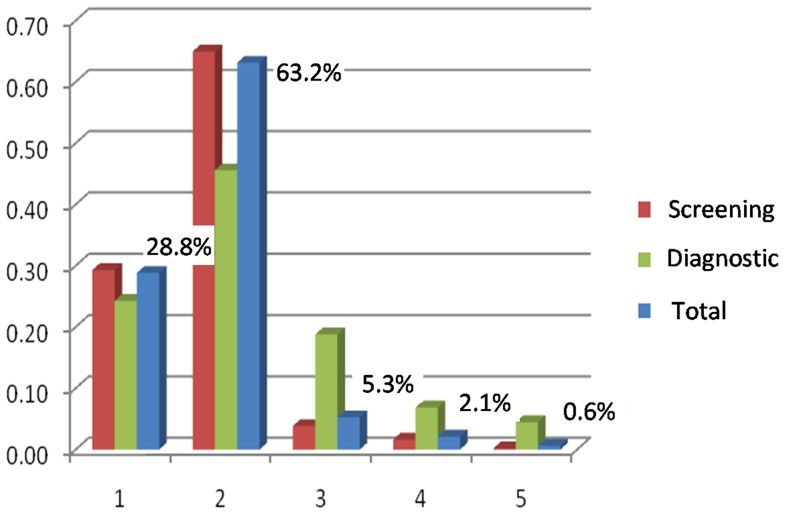
Final BI-RADS distribution (%). Red – screening; Green – diagnostic; Blue – total.

In the entire population, the final BI-RADS classification (Figure 1) included 6905 (92%) benign findings (BI-RADS categories 1 and 2), 396 (5.3%) potentially benign cases (BI-RADS category 3) and 207 ( 2.7%) suspicious and highly suspicious findings (BI-RADS categories 4 and 5).

In the screening mammography (6795), the final BI-RADS classification included 6407 (94.3%) benign findings (BI-RADS categories 1 and 2), 262 (3.9%) potentially benign cases (BI-RADS category 3) and 126 (1.8%) suspicious and highly suspicious findings (BI-RADS categories 4 and 5).

In the diagnostic mammography (713), the final BI-RADS classification included 498 (69.8%) benign findings (BI-RADS categories 1 and 2), 134 (18.8%) potentially benign cases (BI-RADS category 3) and 81 (11.4%) suspicious and highly suspicious findings (BI-RADS categories 4 and 5).

According to the initial mammography, 534 (466 screening and 68 diagnostic) of the 7508 women (7.1%) were classified as BI-RADS category 0 (zero), and these individuals were reclassified after complementation with additional mammographic views and/or ultrasound exams. Of these 534 patients, 455 cases (416 diagnostic screening and 39) (85.2%) were reclassified as BI-RADS categories 1 and 2, 72 cases (46 and 26 diagnostic screening) (13.5%) were classified as BI-RADS category 3 and 7 cases (4 screening and 3 diagnostic) (1.3%) were classified as BI-RADS categories 4 and 5.

### Family history

A total of 65 cases (0.9%) had a positive family history (first degree relatives), 0.8% cases (51 of 6795) were subjected to screening mammography and 2.0% cases (14 of 713) underwent diagnostic exams.

### Biopsies

Regarding the non-surgical biopsies in the screening exams, 19 CNB, 40 FNAB, 5 ultrasound and 72 stereotactic-guided mammotome procedures were performed. Among these procedures, three women had more than one procedure performed at the same facility, and only one individual received malignancy confirmation. This same patient had a final BI-RADS category 5 mammography and opted for treatment at another facility.

Of the diagnostic mammographies, 52 CNB, 23 FNAB and 17 guided mammotome procedures were performed. Among these procedures, seven women underwent more than one procedure at the same facility, and six women obtained malignancy confirmations.

A total of 63 FNAB were performed; 40 tests (63.5%) were screening exams, and only three tests (7.5%) were positive for cancer. In another 23 (36.5%) diagnostic exams, six (26.09%) tests were positive for cancer, one (4.35%) test was a pre-malignant finding and 16 (69.57%) tests were benign findings.

In total, 71 BAGs were performed, 19 BAGS (26.8%) were screening exams and 52 (73.2%) BAGS were diagnostic tests. In the screening tests, 11 (57.9%) tests were positive for cancer, one (5.3%) test was a pre-malignant finding and seven (36.8%) tests were benign findings. Regarding the diagnostic exams, 36 (69.2%) exams were positive for cancer and 16 (30.8%) exams were benign findings.

From the 89 stereotactic-guided mammotome procedures, 72 tests were screening exams and 17 exams were diagnostic tests. In the screening tests, six cases (8.3%) were positive for cancer, one case (1.4%) was DCIS, three cases (4.2%) were pre-malignant findings and 62 cases (86.1%) were benign findings. In the diagnostic tests, one case (5.9%) was positive for cancer, two cases (11.8%) were pre-malignant findings and 14 cases (82.4%) were benign findings.

The five ultrasound-guided mammotomes were all screening exams. No positive cases were observed, one case (20%) was pre-malignant and four cases (80%) were benign findings.

Of the 228 non-surgical biopsies (136 in screening tests and 92 in diagnostic) performed in the service (FNAB, CNB and mammotome), 156 (68.4%) biopsies were benign findings, 63 (27.6%) biopsies were malignant findings, 8 (3.5%) biopsies were pre-malignant findings and one (0.5%) biopsy was DCIS (*in situ* ductal carcinoma).

A total of 162 women with final BI-RADS categories 4 and 5 were subjected to nonsurgical biopsies (96 screening and 66 diagnostic), 51 cases (31.5%) were malignant findings, 102 cases (63%) were benign findings, 8 cases pre-malignant (4.9%) and one case (0.6%) was DCIS (Table 1).

**Table 1:** Biopsy distribution and surgery results.

	Screening (%)	Diagnostic (%)	Total (%)
**Non-surgical biopsies BI-RADS categories 4 and 5**			
Benign	75 (78,1)	27 (40,9)	102 (63,0)
Pre-malignant	5 (5,2)	3 (4,5)	8 (4,9)
Malignant	15 (15,6)	36 (54,5)	51 (31,5)
DCIS	1 (1,1)	0 (0)	1 (0,6)
**Surgical biopsies BI-RADS categories 4 and 5**			
Benign	10 (62,6)	9 (52,9)	19 (57,6)
Malignant	3 (18,7)	7 (41,2)	10 (30,3)
DCIS	3 (18,7)	1 (5,9)	4 (12,1)
**Surgeries**			
Benign	7 (22,6)	9 (17,6)	16 (19,5)
Pre-malignant	4 (12,9)	1 (2,0)	5 (6,1)
Malignant	15 (48,4)	40 (78,4)	55 (67,1)
DCIS	5 (16,1)	1 (2,0)	6 (7,3)

Of the 77 surgeries performed on women with final BI-RADS categories 4 and 5 (28 screening and 49 diagnostic tests), 33 (42.9%) procedures were surgical biopsies (16 screening and 17 diagnostic tests). Of these biopsies, 14 (42.4%) cases had confirmed malignancy and, among these, four cases (12.1%) were ductal carcinoma *in situ* (Table 1).

### Surgeries

When evaluating the anatomopathological results from surgeries from a total of 82 surgeries (31 screening exams and 51 diagnostic exams), 55 (67.1%) procedures were malignant findings, six (7.3%) procedures were DCIS, five (6.1%) procedures were pre-malignant findings (atypical ductal hyperplasia and lobular carcinoma *in situ*) and 16 (19.5%) procedures were benign findings (Table 1).

### Positive cases (BI-RADS 4 and 5) without surgery or biopsy

Cases with final positive BI-RADS were not biopsied, and we opted for early mammographic follow-up. A total of 18 cases (8.7%) were obtained, 13 of these cases were (72.2%) stable screening exams during the 2 years of follow-up, and five (27.8%) cases were diagnostic tests. Of these diagnostic tests, four (BI-RADS category 4A) exams were stable after two years and one exam was classified as BI-RADS category 5 (by palpable focal asymmetry) at early follow-up, revealing a reduction in the dimensions of these cancers. After collecting the details of this last case, we observed that the patient had undergone the first examination during the premenstrual period.

A total of 19 additional cases with BI-RADS categories 4 and 5 (9.2%) were not followed in the service. Of these, 12 (63.2%) cases were screening tests and seven (36.8%) were diagnostic tests. The patients were examined, but the contact with ten of these patients was not possible (these individuals could not be contacted at the phone number provided). Among the contacted individuals, 2 patients underwent treatment at another facility, four patients reported benign biopsies collected at another hospital, one (1) individual refused treatment after the diagnosis of breast cancer and one individual died of unknown causes (not informed by relatives) before the initiation of the procedures.

### Association measures

In the true-positives exams (TP), 29 (44%) individuals were affected in the right breast, 35 (53%) individuals were affected in the left breast and two (3%) patients were affected in both breasts.

In the 66 true-positive cases (TP), the cancer detection rate was 8.8 per 1000 cases. In the screening exams, 22 cases were TP and the detection rate was 3.2 cancers per 1000 cases. In diagnostic exams, 44 cases were TP, and the cancer detection rate was 61.7 in 1000 cases (Table 2).

**Table 2:** Final BI-RADS classification and detected cancer.

	Positive Cancer		Negative Cancer		
	Screening	Diagnostic	Screening	Diagnostic	Total
Positive mammogram (BI-RADS categories 4 and 5)	22	44	104	37	207
Negative mammogram (BI-RADS categories 1, 2 and 3)	3	2	6666	630	7301
Total	25	46	6770	667	7508

P<0,01.

Sensibility - Screening: 88%; Diagnostics: 95,7%; Total: 93%.

Specificity - Screening: 98,5%; Diagnostic: 94,4%; Total: 98,1%.

Of the cancers diagnosed and treated at the service, seven (11.1%) were DCIS, and 56 (88.9%) were invasive carcinomas. Three cases of cancer were not followed at the service.

In the screening exams (n  =  21), six (28.6%) cases were DCIS and 15 (71.4%) cases were invasive carcinomas. In diagnostic tests, one case (2.4%) was DCIS and 41 (97.6%) cases were invasive carcinomas. Four cases of breast cancer were not followed at the service: the results for three of these cases were not assigned by patients and in the other case, the patient refused treatment. These four cases had initial negative mammograms.

The global recall rate was 9.7% (730 of 7508). The PPV1 (percentage of cancer after positive mammogram with BI-RADS categories zero, 4 and 5) was 9% (66 of 730), PPV2 (percentage of cancers determined by screening exams with final BI-RADS categories 4 and 5) was 31.9% (66 of 207), and PPV3 (percentage of cancers after biopsy of cases with BI-RADS categories 4 and 5 and positive biopsy rates) was 37.7% (66 of 175) (Table 3).

**Table 3:** General results from 7508 mammographies.

	TP	FP1	FP2	FP3	Positive mammogram (0,4,5)	PPV1	PPV2	PPV3	Detection rate	Recall rate
Screening	22	564	104	80	586	3,7	17,5	21,6	3,2	8,6
Diagnostic	44	100	37	29	144	30,6	54,3	60,3	61,7	20,2
Total	66	664	141	109	730	9,0	31,9	37,7	8,8	9,7

In screening tests, the recall rate was 8.6% (586 of 6795). The PPV1 was 3.8% (22 of 586), the PPV2 was 17.5% (22 of 126) and PPV3 was 21.6% (22 of 102) (Table 2).

In diagnostic tests, the recall rate was 20.2% (144 of 713). The PPV1 was 30.6% (44 of 144), the PPV2 was 54.3% (44 of 81) and PPV3 was 60.3% (44 of 73) (Tables 4 and 5).

**Table 4:** Cancer detection in the total mammographies evaluated.

Detected cancer characteristics	Screening n (%)	Diagnostic n (%)	Total (7508) n (%)
Tumor type			
DCIS	6 (28,6)	1 (2,4)	7 (11,1)
Invasive	15 (71,4)	41 (97,6)	56 (88,9)
Minimal cancer (DCIS and invasive cancer ≤10 mm)	11 (52,4)	4 (9,5)	18 (28,6)
Nodal invasion			
Yes	5 (23,8)	25 (59,52)	30 (47,6)
No	16 (76,2)	17 (40,48)	33 (52,4)
Unknown	1 (4,5)	2 (4,5)	3 (4,5)
Stage*			
0	5 (23,8)	1 (2,4)	6 (9,5)
I	8 (38,1)	3 (7,1)	11 (17,5)
II	6 (28,6)	22 (52,4)	28 (44,4)
III	1 (4,8)	12 (28,6)	13 (20,6)
IV	1 (4,8)	4 (9,5)	5 (7,9)
Unknown	1 (4,5)	2 (4,5)	3 (4,5)

Note: The number of all detected cancers was 66 and the detection rate was 8.79 per 1,000.

Screening exams - detected cancers: 22; detection rate of 3.24/1000.

The percentage of all cancers diagnosed as DCIS was 10.6% (7 of 66). Of all invasive cancers with defined dimensions, 17.5% (11 of 63) of these cancers were less than or equal to 10 mm. The percentage of cancers considered minimal (DCIS and smaller than 10 mm invasive cancers) was 28.6% (18 of 63) (Table 3).

In the screening exams, the percentage of cancers diagnosed as DCIS was 27.3% (6 of 22). Of all invasive cancers with defined dimensions, 53.3% (8 of 15) were less than or equal to 10 mm. The percentage of cancers considered minimal (DCIS and smaller than 10 mm invasive cancer) was 52.4% (11 of 21). (Table 3)

In the diagnostic exams, the percentage of DCIS in diagnosed cancers with known histological types was 2.4% (1 of 42). Of all invasive cancers with defined dimensions, 7.3% (3 of 41) were less than or equal to 10 mm. The percentage of cancers considered minimal (DCIS and smaller than 10 mm invasive cancer) was 9.5% (4 of 42). (Table 2)

The cases with nodal invasions were 47.6% in total (30 of 63), all with known nodal statuses. Of the cancers with known stages, 27% (17 of 63) were classified as stage 0 (zero) and I. The percentage of cancers with insufficient information to calculate the staging was 4.5% (3 of 66). (Table 2)

In the screening exams, 23.8% (5 of 21) of the patients showed nodal invasion, all with known nodal status. Of the cancers with known stages, 61.9% (13 of 21) were classified as stage 0 (zero) and I. The percentage of cancers with insufficient information to calculate the staging was 4.5% (1 of 22). (Table 2)

In the diagnostic exams, 59.5% (25 of 42) of the patients showed nodal invasion, all with known nodal status. Among the cancers with known stages, 9.5% (4 of 42) were classified as stage 0 (zero) and I. The percentage of cancers with insufficient information to calculate the staging was 4.5% (2 of 44). (Table 2)

The sensitivity and specificity of the mammographies performed during the specified period were 93% and 98.1%, respectively (Table 3)

### Survival analysis

**Figure 2:**
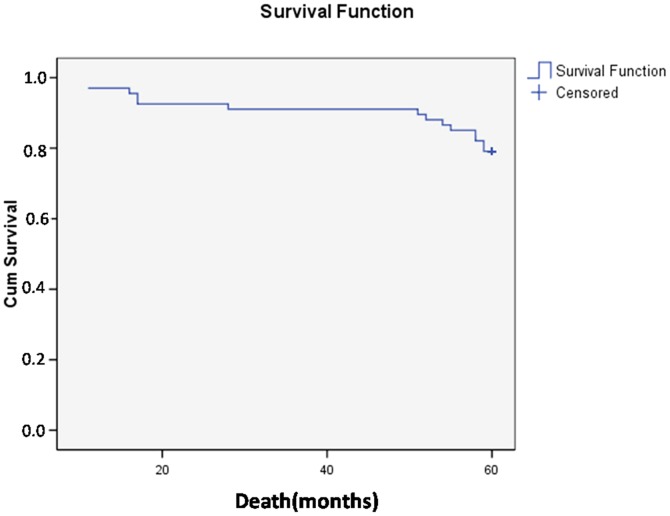
Survival curve (Kaplan-Meier) of the patients in the study.

Among the 71 patients with breast cancer, 67 (94.4%) patients were followed for a period of five years after diagnosis. Four patients were not included because they sought treatment at other facilities and data was not available. The total number of deaths in the followed cases was 14. There was no statistically significant predominance of deaths among the cancer staging. Only three patients remained alive with recurrent cancer, representing 5.7% of live patients and 60% (3/5) of recurrent cancer cases. The overall survival rate after five years was 79.1% (Figure 2)

In the present study, we observed that the larger the tumor, the greater the relative risk of death from breast cancer.

## Discussion

This study evaluated the introduction of a pilot program for the early detection of breast cancer in a defined population.

In Brazil and other developing countries, mammography-screening programs are primarily opportunistic rather than used to screen asymptomatic women [11], [12]. The Brazilian National Cancer Institute (INCA) formerly recommended that all women between 40 and 49 years of age should have clinical breast examination, and women aged 50 to 69 years should have a mammogram every 2 years [1]. However, since 2009, federal laws have made it mandatory for women aged 40 years and older to receive annual mammograms.

The studies used as the basis for planning the health policies in Brazil primarily originate from developed countries, where the health systems and population characteristics are different from those of Brazil. Thus, we propose that low- and middle-income countries, such as Brazil, should utilize their own breast cancer data to develop national health policies towards increased efficacy, *e.g.* in the breast cancer screening programs.

In this study, we observed that a higher percentage of young women with breast cancer (40–49 y/o), likely reflecting the high prevalence of young people in that area. In another study, in the region of Barretos, SP, Brazil, higher rates of breast cancer in younger women were also observed, demonstrating the importance of early mammographic screening in this region [13].

The breast cancer detection rate is associated with the incidence and prevalence of disease in a population, the characteristics of the screened population (mean age, breast density, previous exposure to screening and risk of breast cancer), the characteristics of the local health system (degree of concern about medico-legal complaints, financial incentives, and quality control) and the quality of service [14]. Methodological parameters might also influence the results reported, such as the inclusion or exclusion of palpable nodules. The detection rate increases with the age of the screened population [15]-[17] and decreases with prior exposure to screening [18]–[19].

In this study, we calculated a detection rate of 3.24/1000 in asymptomatic patients, 61.71 in symptomatic patients and 8.79 in the total exams. These detection rates were consistent with previously published studies. Sickles et al. [20] achieved a detection rate of 25.3/1,000 in symptomatic patients. Keen and Keen [16] showed a detection rate of 1.9/1000 at age 40, a rate of 7.2/1000 at age 50 and a rate of 15.1/1000 at age 60. Smith-Bindman et al. [17] compared with the results from three large screening programs, including two programs in the United States (BCSC, NBCCEDP) and one program in England (NHSBSP), analyzing 5.5 million mammograms in patients 50 years or older; the results showed detection rates of 5.8, 5.9, and 6.3/1000, respectively. Jiang et al. [16] conducted a multicenter study using 510 radiologists to analyze the results of 2,289,132 screening mammograms, showing a detection rate of 3.91/1000. The BI-RADS considers a detection rate between two and ten as desirable. A rate of six to ten patients has been observed for individuals undergoing their first mammography, and a rate of two to four patients for individual who have been previously screened [8].

Sohlich et al. [9] observed that in mixed services (screenings and diagnoses evaluated together), in a proportion of 90 diagnostic exams per ten screening exams, the detection rate should be ten. The current study showed that the proportion of screening/diagnostic exams resulted in a lower detection rate, potentially reflecting the large number of patients younger than 50 years.

The positive predictive value indicated in this work was 9.04% between all mammograms considered as abnormal (PPV1) and 31.88% among the patients who received biopsy indications (VPP2). The BI-RADS considers a PPV1 of 5–10% and PPV2 of 25–40% as desirable. In a multicenter study, Taplin et al. [20] reported a PPV1 of 4.1% and PPV2 of 38.8%. Duijm et al. [21] observed a PPV2 of 37.4%. Rosenberg et al. [22] observed a PPV1 of 4.8% and a PPV2 of 24.6%. In a Brazilian study, Azevedo et al. [23] reported a PPV1 of 11% and a PPV2 of 31%.

The recall rate in this study was 9.72% in the total population, 8.62% in the screening population and 20.20% in the diagnostic population. Other authors reported 14.2 to 15.7% (England) [15], 9.8% (USA) [24] and 2.7% (Norway) [24], 14.4% and 12.5% (USA) and 7.6% (England) [17]. Schell et al. [25] estimated that the recall rates leading to optimal results would be 10% in the first mammogram and 6.7% in subsequent mammograms. The BI-RADS considers a recall rate of less than 10% as desirable [8].

Variations in the accuracy and results of mammography have been observed. In Brazil, data concerning the population are scarce. In June 2009, the SISMAMA [26] was established to manage breast cancer early detection programs, but the system remains flawed. In a screening program for breast cancer, the ratio of invasive carcinoma to carcinoma *in situ* represents the frequency of invasive lesions to noninvasive lesions between the identified cancers. This ratio increases with increasing age, suggesting that higher ratios are observed in older age groups. In the analyzed time period, the ratio for all the age groups in SISMAMA was 9.0; a ration of 2.7 was observed in the present study. This result confirms that breast cancer is diagnosed late in Brazil.

The BI-RADS showed that more than 30% of cancers diagnosed in screening exams are minimal (DCIS and invasive less than 1.0 cm) and more than 50% of the cancers are in the early stages (zero and I) [8]. In this population sample, we detected 28.6% minimal cancers and 27% of cancers in the early stages. Considering only screening exams, we detected a significantly higher detection rate of 53.3% minimal breast cancers and 61.9% early stage cancers. Compared with other studies [19], [27] the rate of cancers at advanced stages was actually higher (90.5% in this sample population) in diagnostic tests. Sohlich et al. [9] showed 90:10 in mixed services, indicating a rate of 87%.

The survival rates at 5 years for breast cancer widely varied between countries. A study published in 2008, reported 5-year rates of 58.4% for breast cancer in Brazil, 83.7% in the U.S., 73.1% in Europe and 81.6% in Japan [26]. Studies with patients treated at INCA-Brazil, showed that breast tumors for the overall survival rate at five years was 52% [27]. The survival rate of 79.1% observed in this study demonstrates that the introduction of a screening and diagnostic mammographic service contributes to an early diagnosis of breast cancer in this population.

There are several limitations that should be addressed in this study. The lack of demographic data precluding comparisons between screened and unscreened groups and the impossibility of monitoring patients that might present different registration numbers at each service, thereby requiring the manual management of this problem for collecting these data. Failures in the Unified Health System in Brazil (UHS) were also observed, making it difficult to collect adequate epidemiological data. In addition, our analyses only controlled for age and tumor stage at diagnosis because data were not available for other potential prognostic factors, such as comorbid diseases. Moreover, most of Brazilian programs are opportunistic, and some authors have reported low attendance rates in their programs [11].

## Conclusions

The evaluation of the breast cancer screenings and diagnostic programs in a geographically defined population at São Paulo does not clarify the complex issues associated with the epidemiology of breast cancer in Brazil. Many challenges still remain, but the data obtained in this study provide considerable information to guide future efforts. Survival is a key index of the overall effectiveness of health services in the management of patients with cancer. Our results suggest that a screening and diagnostic program is feasible and can potentially improve breast cancer outcomes for many women in São Paulo. We propose that these data will also contribute to the development of similar programs in other areas of Brazil and other developing countries with similar socioeconomic statuses.
