# Correction: Mammographic Assessment of a Geographically Defined Population at a Mastology Referral Hospital in São Paulo Brazil

**DOI:** 10.1371/journal.pone.0351091

**Published:** 2026-06-04

**Authors:** Simone Caetano, Juvenal Mottola Junior, Flora Finguerman, Suzan M. Goldman, Jacob Szejnfeld

In the Sample population subsection of the Methods, a reference is missing from the third sentence of the seventh paragraph.

The correct sentence is: The average household income of this population was approximately $ 390 USD, while in the city of Sao Paulo the average income is $ 663 USD, with a high prevalence of people under 40 years of age (Fundação SEADE, 2024).

The reference is: Fundação Sistema Estadual de Análise de Dados (SEADE). Indicadores socioeconômicos do Município de São Paulo: SEADE. Available from: https://produtos.seade.gov.br.
